# Supplementary material for: Participation of Extracellular Vesicles from Zika-Virus-Infected Mosquito Cells in the Modification of Naïve Cells’ Behavior by Mediating Cell-to-Cell Transmission of Viral Elements
Source: Cells. 2020 Jan 4;9(1):123. doi: 10.3390/cells9010123 (PMC7016930; doi:10.3390/cells9010123)
Supplement: Supplementary file 1 [file cells-09-00123-s001.pdf]

Supplementary Materials

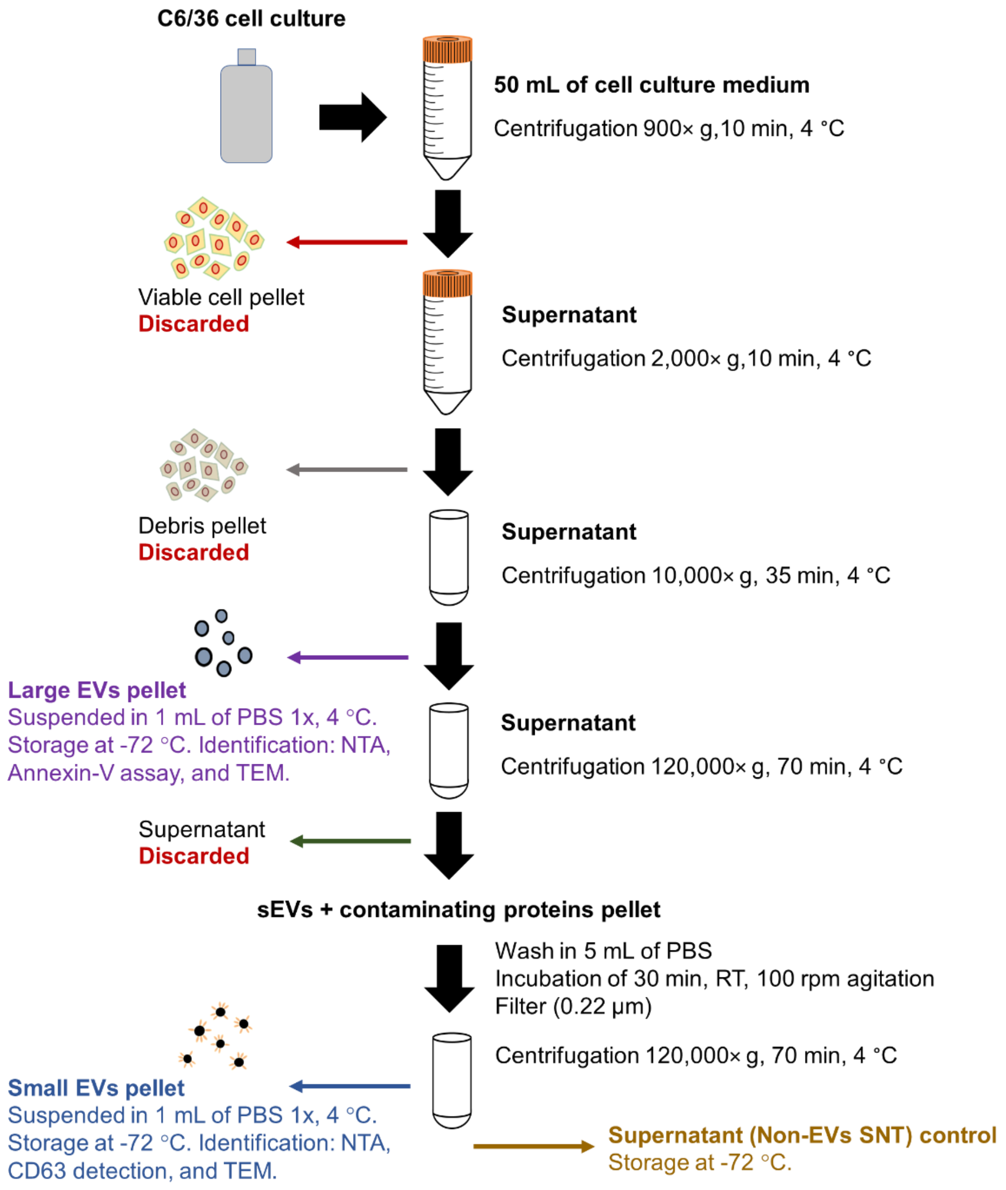

Figure S1. Graphic description of the EVs isolation by ultracentrifugation. NTA = Nanoparticles tracking analysis; TEM = Transmission electron microscopy; rpm = revolutions per minute.

A

Parameter	Evaluated conditions	Optimal conditions
Camera level	10, 12, 14, 16	12
Detection threshold	2.0, 2.5, 5.0	2.5
Temperature (°C)	15, 20, 25	20
Dilution	1:25, 1:50, 1:100	1:50
Diluent	PBS 1x, Water	PBS 1x
Load volume (mL)	0.5, 1.0, 1.5	1.0
Reading times (s)	30, 60, 90	30
Repetitions	2, 3, 5	3

Nanoparticles Tracking Analysis (NTA)

B

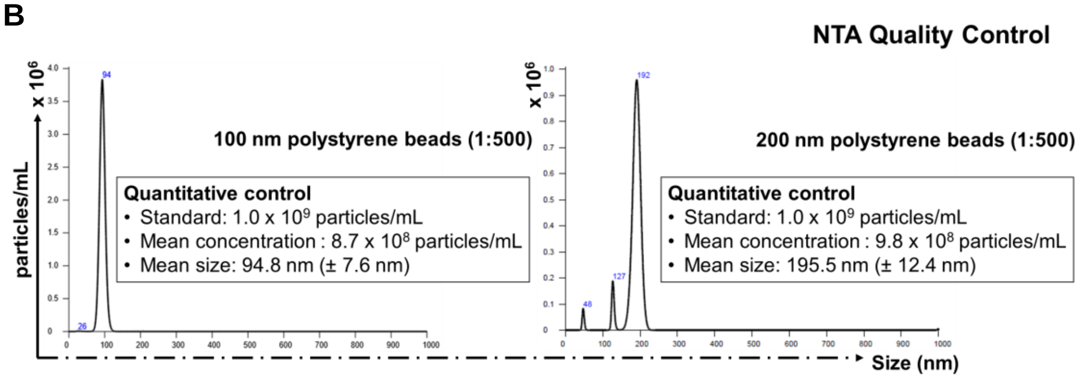

C

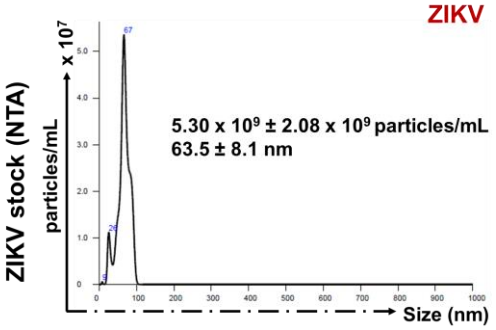

Figure S2. Nanoparticles Tracking Analysis (NTA) (**A**) Description of the evaluated and optimal conditions for nanoparticles quantification by NTA. (**B**) NTA quality control based on the optimal conditions established. Histograms are the representative of three independent experiments using polystyrene beads (100 and 200 nm) diluted at 1:500. (**C**) NTA of purified ZIKV stock. Histogram is the representative mean ± SD of nanoparticles concentration (particles/mL) and size (nm) from three independent experiments.

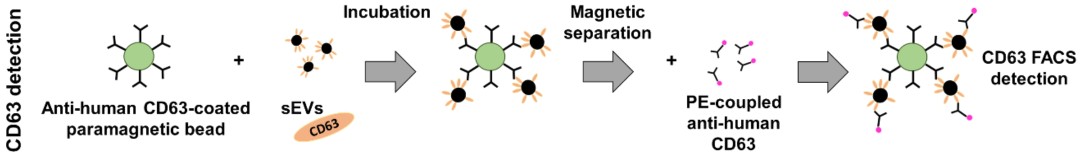

Figure S3. Detection of SEVs CD63+. Graphic description of sEVs CD63+ detection by magnetic separation with paramagnetic beads coated with anti-CD63.

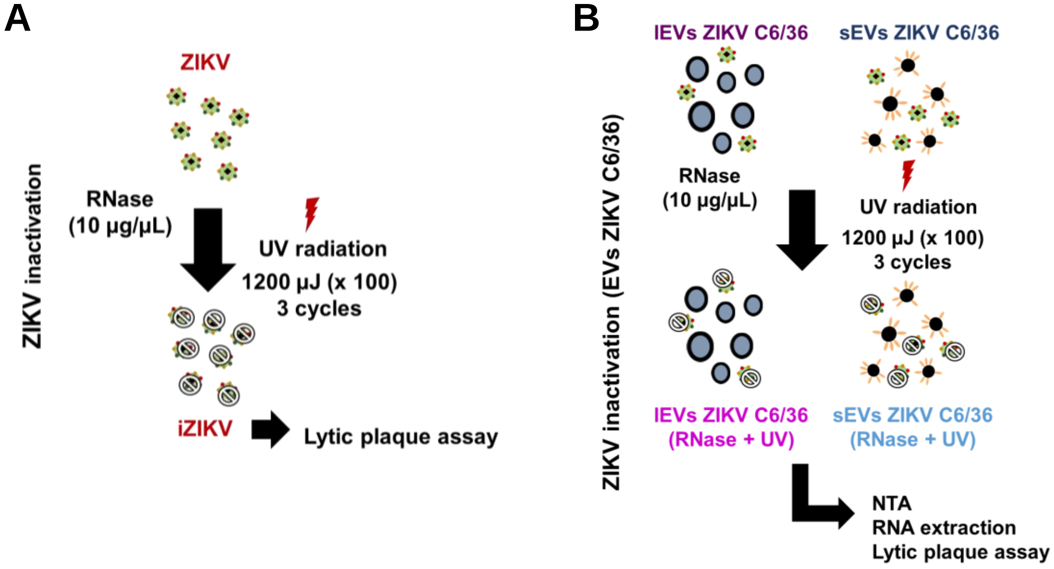

Figure S4. ZIKV inactivation. (**A**) Graphic description of RNase A assay and UV irradiation for ZIKV inactivation. iZIKV = inactivated ZIKV. (**B**) Graphic description of RNase A assay and UV irradiation for ZIKV inactivation on ZIKV-infected C6/36 EVs isolates.

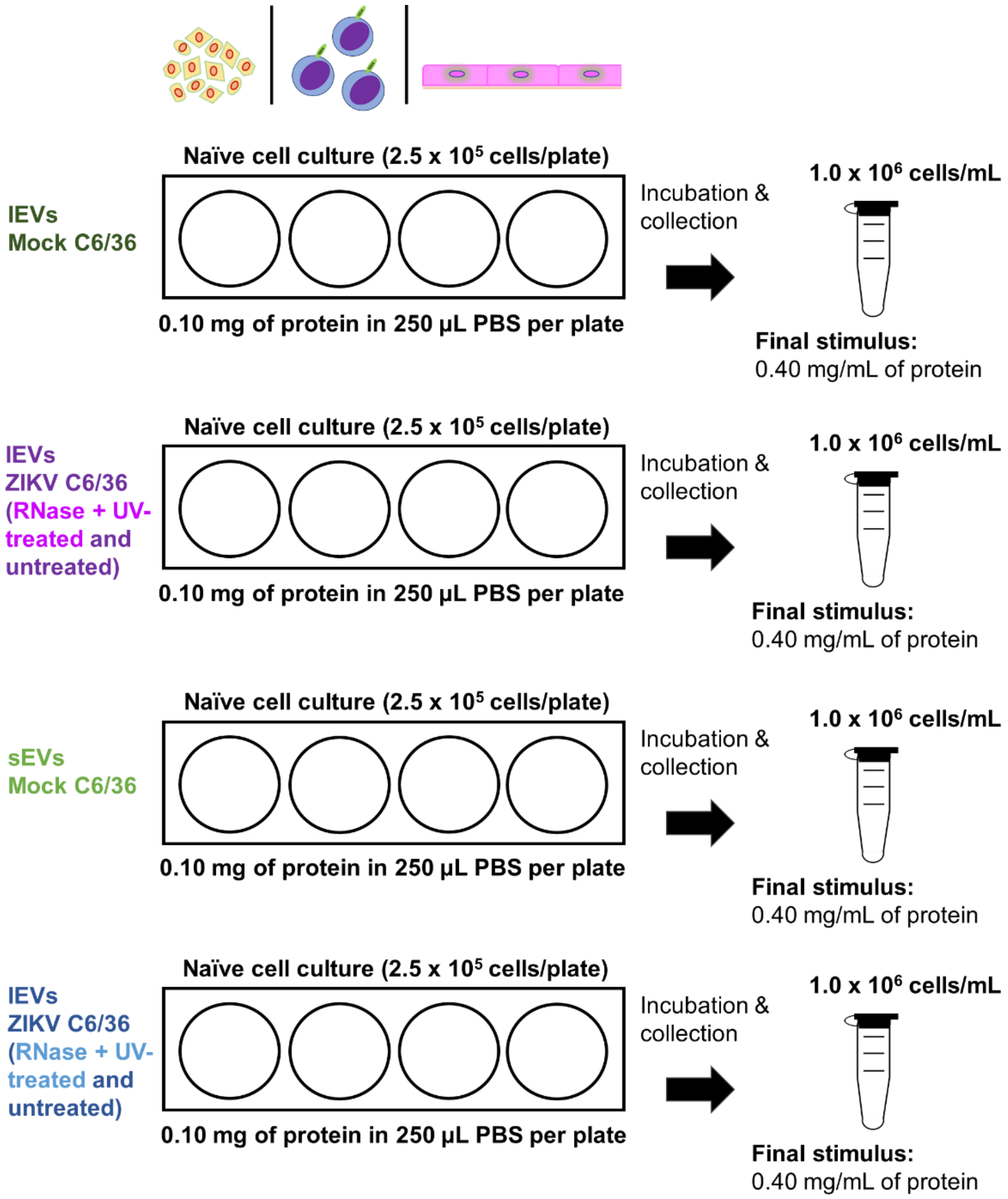

Figure S5. Graphic description of the EVs stimulation assays on naïve cells (C6/36, THP-1, or HMEC-1).

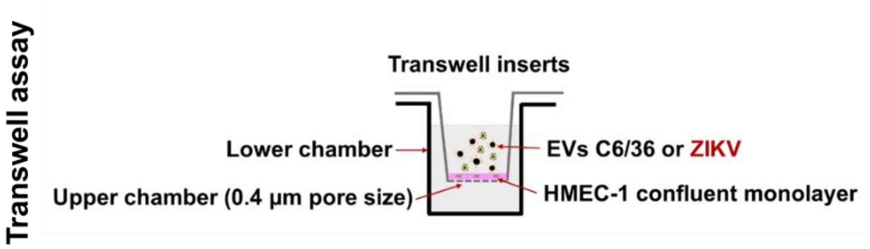

Figure S6. Vascular endothelial permeability assay. Graphic description of the Transwell system used in the vascular endothelial cells permeability assay.

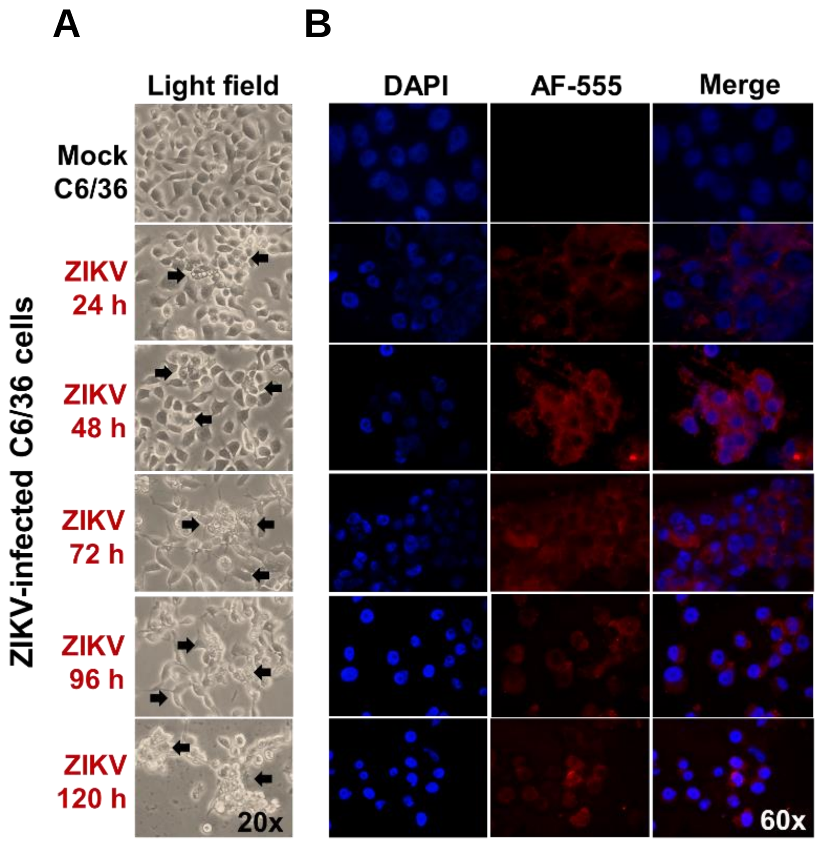

Figure S7. Microscopies from ZIKV-infected C6/36 cells. (**A**) Cytopathic effects observation at 24, 48, 72, 96, and 120 h PI (black arrows) evaluated by light-field microscopy (20x). (**B**) ZIKV E protein detection (red) by fluorescence microscopy (60x).

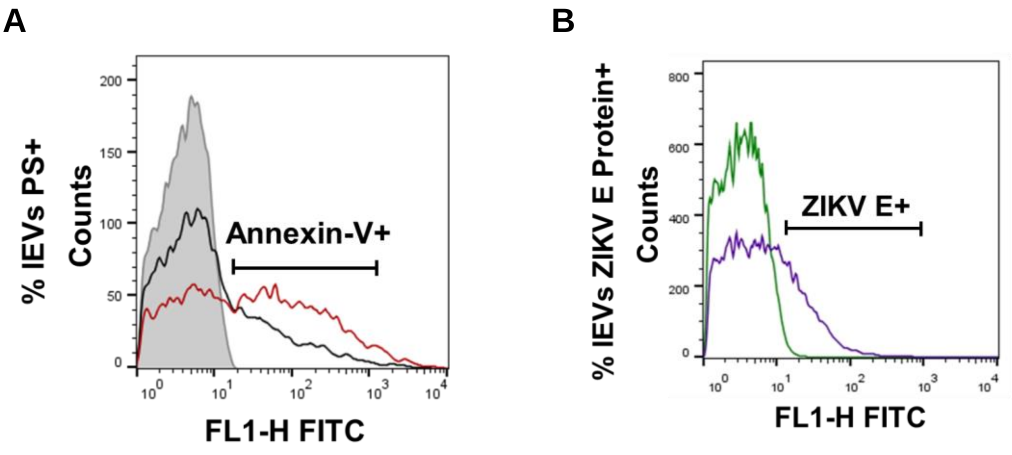

Annexin-V

	MFI	SD
Beads	4.86	0.06
Mock	219.50	3.61
ZIKV	318.67	6.50

ZIKV E Protein

	MFI	SD
lEVs Mock C6/36	5.02	0.20
lEVs ZIKV C6/36	93.53	2.06

Figure S8. Mean fluorescence intensity (MFI) values from FACS assays performed on lEVs isolates. (**A**) Annexin-V binding MFI values. Histograms are the representative of lEVs PS+ distribution from three independent experiments. (**B**) ZIKV E Protein MFI values. Histograms are the representative of lEVs ZIKV E Protein+ distribution from three independent experiments.

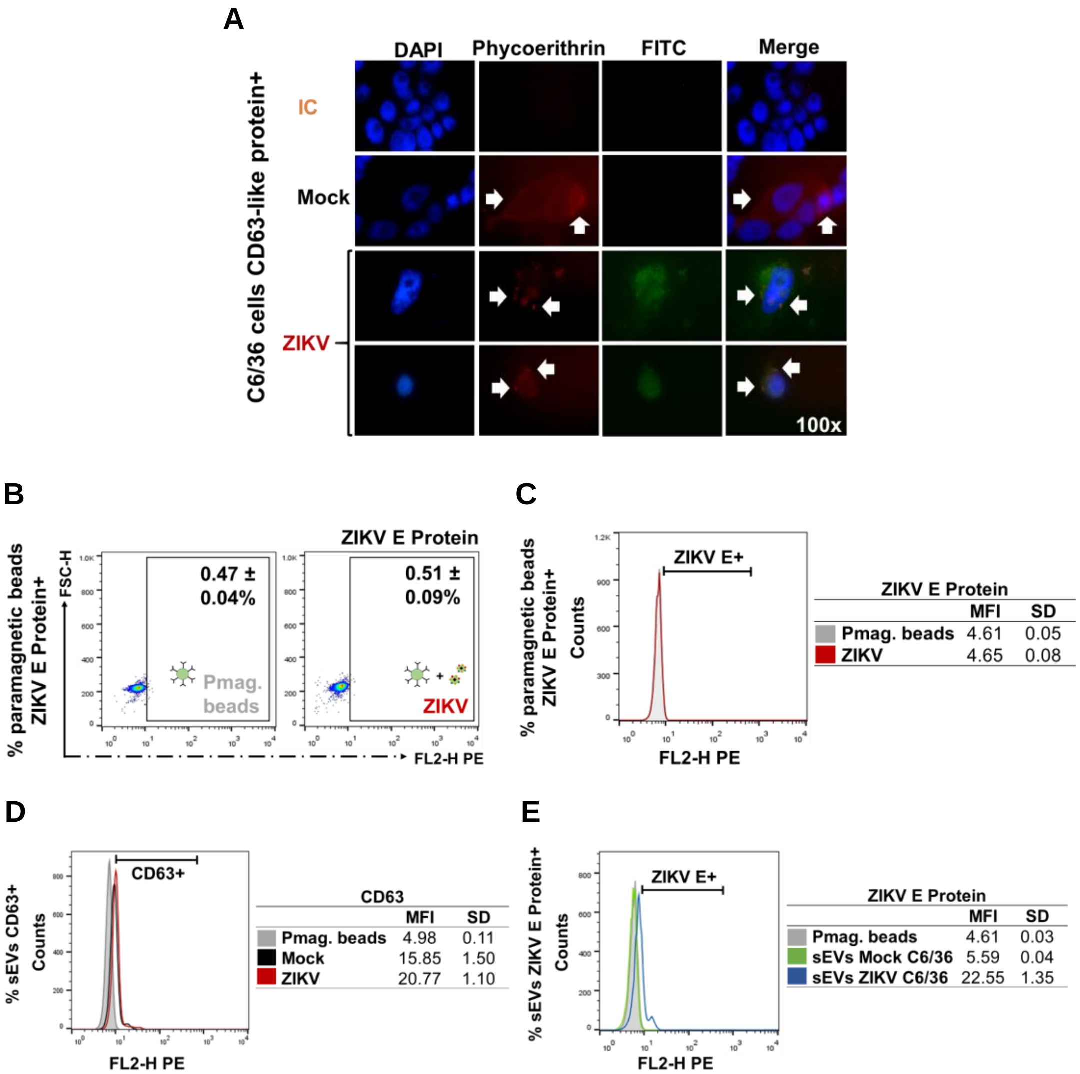

Figure S9. Identification of small EVs CD63+ from C6/36 cells. (**A**) CD63-like protein detection (red) by fluorescence microscopy (100x) in the Mock and ZIKV-infected cells (green for ZIKV E protein). (**B**) Detection of ZIKV coupled to paramagnetic beads coated with anti-CD63 by FACS. Dot plots are the representative mean ± SD of the ZIKV E Protein+ (ZIKV coupled) from three independent experiments. (**C**) ZIKV E Protein+ MFI values from ZIKV coupled to paramagnetic beads. Histograms is the representative of the ZIKV E Protein+ distribution from three independent experiments. (**D**) CD63 MFI values. Histograms are the representative of sEVs CD63+ distribution from three independent experiments. (**E**) ZIKV E Protein MFI values. Histograms are the representative of sEVs ZIKV E Protein+ distribution from three independent experiments.

A

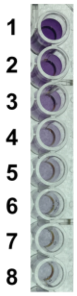

	Concentration (mg/mL)	Absorbance
1	2.00	1.413
2	1.00	0.998
3	0.50	0.581
4	0.25	0.439
5	0.125	0.373
6	0.0625	0.323
7	0.03125	0.303
8	0.015625	0.285

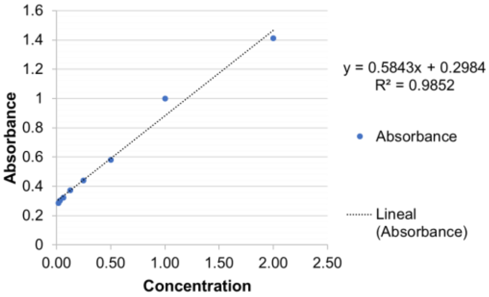

B

	lEVs Mock C6/36		lEVs ZIKV C6/36		sEVs Mock C6/36		sEVs ZIKV C6/36	
	Mean (Absorbance)	Concentration (mg/mL)	Mean (Absorbance)	Concentration (mg/mL)	Mean (Absorbance)	Concentration (mg/mL)	Mean (Absorbance)	Concentration (mg/mL)
Pool 1	0.579	**0.480**	0.592	**0.502**	0.585	**0.491**	0.608	**0.530**
Pool 2	0.585	**0.491**	0.598	**0.512**	0.599	**0.514**	0.612	**0.537**
Pool 3	0.589	**0.497**	0.602	**0.520**	0.582	**0.485**	0.625	**0.545**

Figure S10. Protein quantification of the EVs isolates. (**A**) Calibration curve obtained by BCA method. (**B**) Protein concentration (mg/mL) from EVs isolates. Values were calculated from the mean of absorbances obtained in three independent measures.

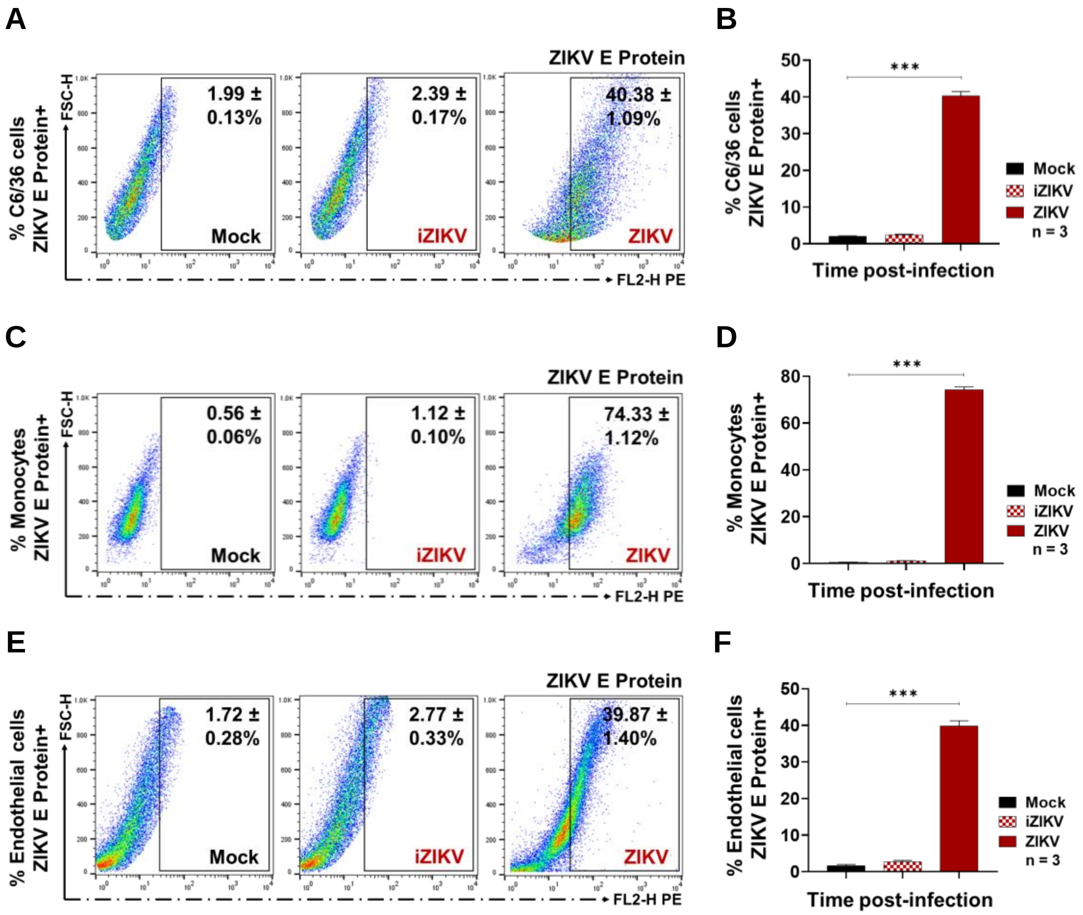

Figure S11. Inactivated ZIKV (iZIKV) does not infect C6/36, THP-1, or HMEC-1 cells. (**A,C,E**) ZIKV E protein detection at 48 h (C6/36), 96 h (THP-1), or 72 h (HMEC-1) PI by FACS assay, respectively. ZIKV stock was used as positive control. Dot plots are the representative mean ± SD of the positive cells from three independent experiments. (**B,D,F**) ZIKV-infected cells percentages obtained by FACS. The ZIKV E protein levels were compared (by unpaired Student's t-test) with the Mock cells (*) value. Statistical significance was recognized as * when $p < 0.05$, ** when $p < 0.01$, and *** when $p < 0.0001$.

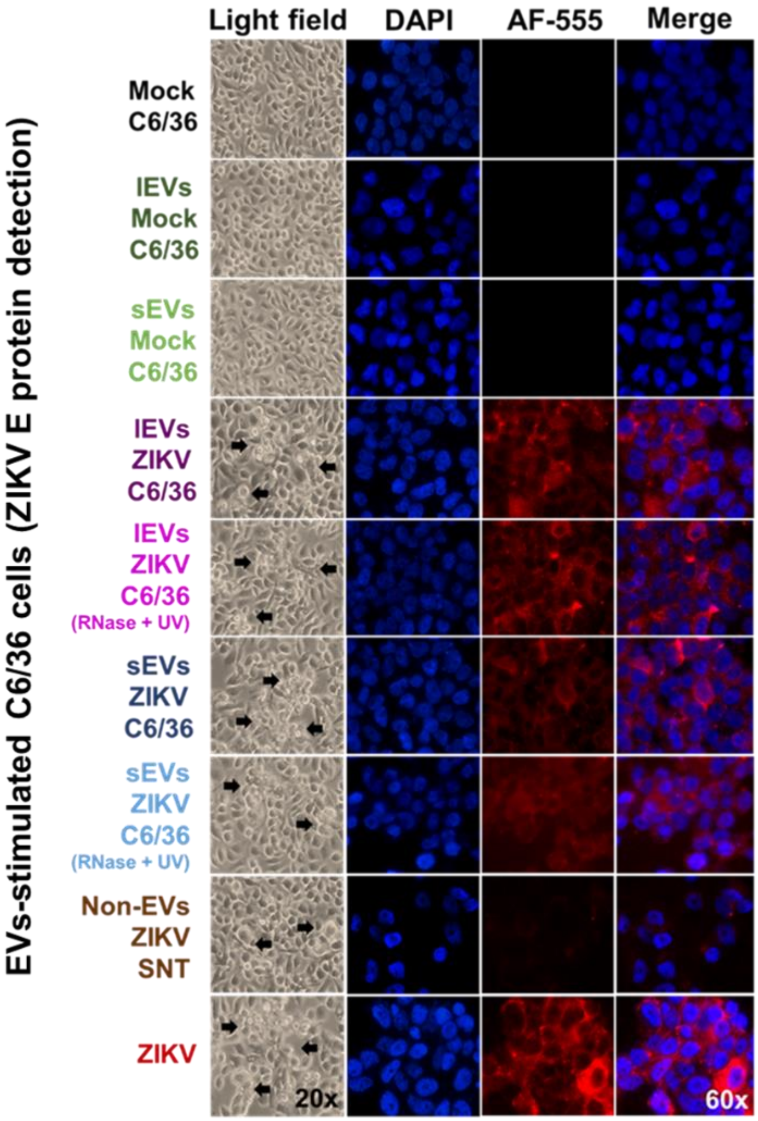

Figure S12. ZIKV C6/36 EVs-stimulated naïve C6/36 cells present ZIKV E Protein on their membranes (48 h PI). Cytopathic effects observation (black arrows) and ZIKV E protein detection (red) evaluated by light-field (20x) and fluorescence microscopy (60x), respectively.

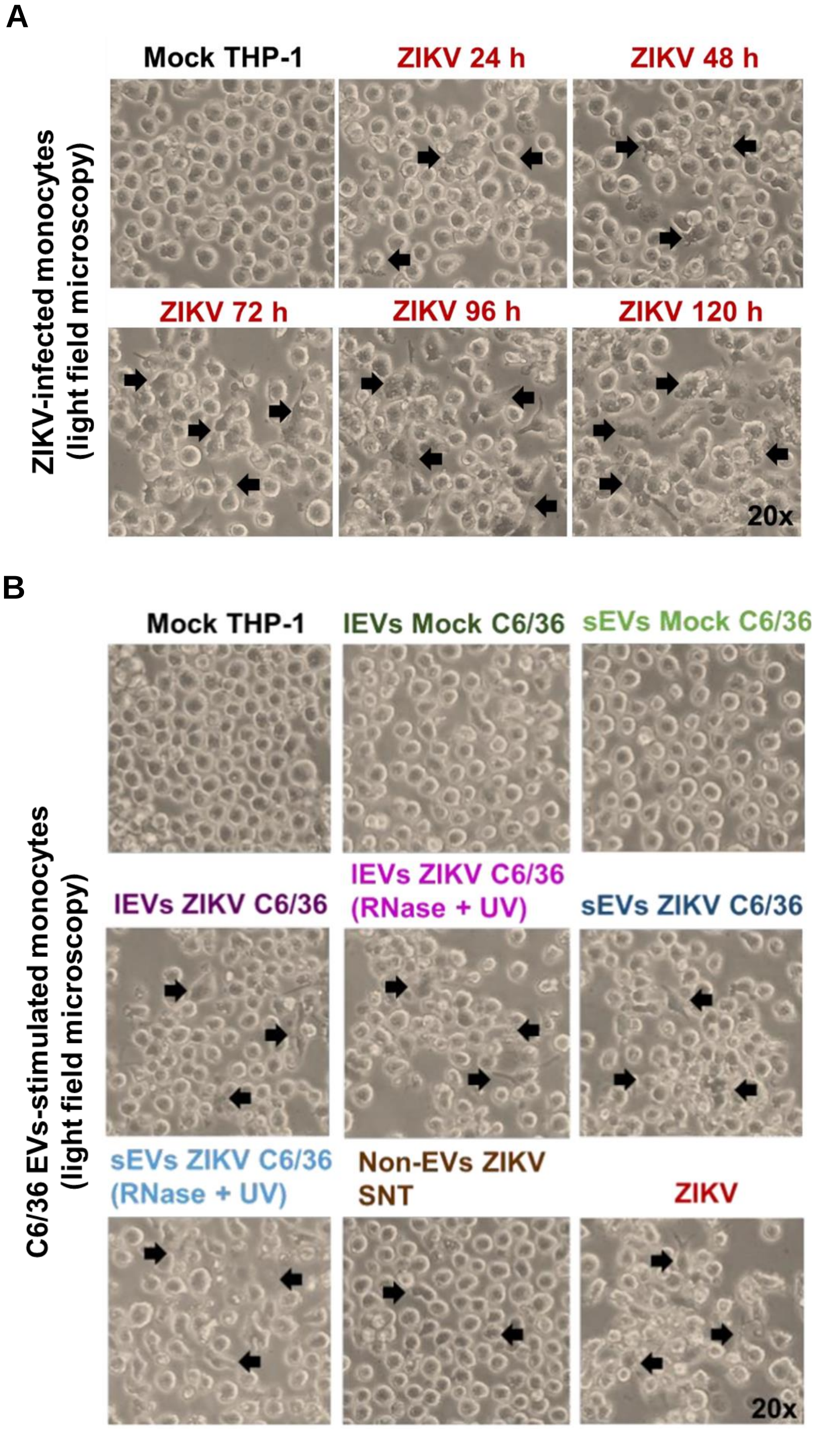

Figure S13. Microscopies from ZIKV-infected and C6/36 EVs-stimulated monocytes. (**A**) Cytopathic effects observation at 24, 48, 72, 96, and 120 h PI (black arrows) evaluated by light-field microscopy (20x). (**B**) Cytopathic effects observation at different EVs stimuli conditions (black arrows) evaluated by light-field microscopy (20x).

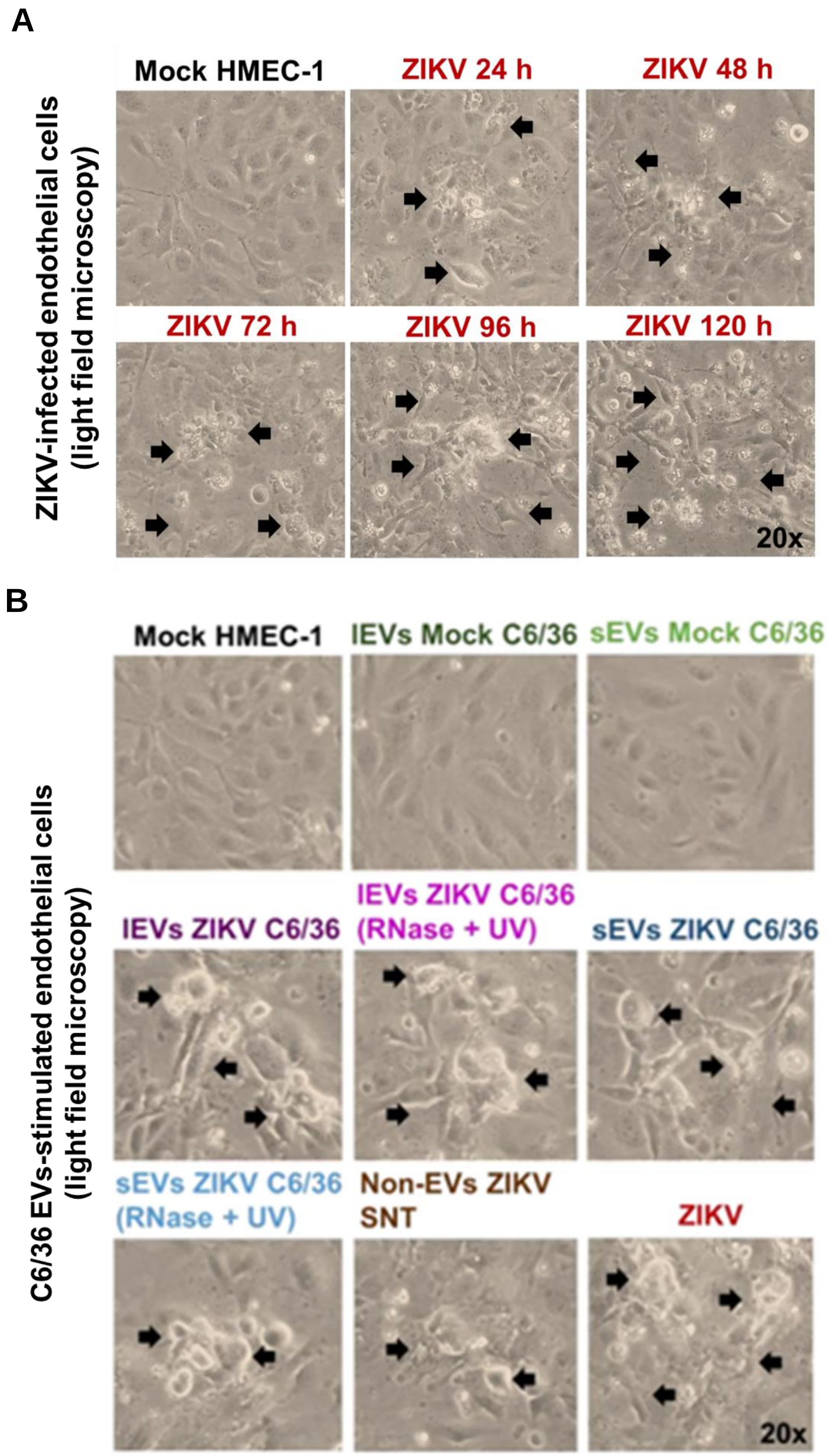

Figure S14. Microscopies from ZIKV-infected and C6/36 EVs-stimulated endothelial cells. (**A**) Cytopathic effects observation at 24, 48, 72, 96, and 120 h PI (black arrows) evaluated by light-field microscopy (20x). (**B**) Cytopathic effects observation at different EVs stimuli conditions (black arrows) evaluated by light-field microscopy (20x).
